# Efficacy of radial styloid targeting screws in volar plate fixation of intra-articular distal radial fractures: a biomechanical study in a cadaver fracture model

**DOI:** 10.1186/1749-799X-5-90

**Published:** 2010-12-02

**Authors:** Kousuke Iba, Yasuhiro Ozasa, Takuro Wada, Tomoaki Kamiya, Toshihiko Yamashita, Mitsuhiro Aoki

**Affiliations:** 1 Department of Orthopaedic Surgery School of Medicine, Sapporo Medical University; 2 Department of Orthopaedic Surgery, Sapporo Daiichi Hospital

## Abstract

**Background:**

The locking screws target the radial styloid, theoretically provide greater stability against radial styloid fragment. However, it is unknown whether the radial styloid locking screws increased the stability of the volar plating system fixation along the entire distal radius or not. In this study, we evaluated the stability of the volar plating system fixation with or without the radial styloid screws using a biomechanical study in a cadaver fracture model.

**Methods:**

Six matched pairs of fresh-frozen human cadaver wrists complete from the proximal forearm to the metacarpal bones were prepared to simulate standardized 3-part intra-articular and severe comminuted fractures. Specimens were fixed using the volar plating system with or without 2 radial styloid screws. Each specimen was loaded at a constant rate of 20 mm/min to failure. Load data was recorded and, ultimate strength and change in gap between distal and proximal fragments were measured. Data for ultimate strength and screw failure after failure loading were compared between the 2 groups.

**Results:**

The average ultimate strength at failure of the volar plate fixation with radial styloid screws (913.5 ± 157.1 N) was significantly higher than that without them (682.2 ± 118.6 N). After failure loading, the average change in gap between the ulnar and proximal fragment was greater than that between the radial and proximal fragment. The number of bent or broken screws in ulnar fragment was higher than that in radial fragment. The number of specimens with bent or broken screws in cases with radial styloid screws was fewer than that in the fixation without radial styloid screws group.

**Conclusion:**

The ulnar fragment is more intensively stressed than the radial fragment under axial loading of distal radius at full wrist extension. The radial styloid screws were effective in stable volar plate fixation of distal radial fractures.

## Background

A number of techniques exist for the treatment of distal radius fractures including closed reduction and cast immobilization, percutaneous pin fixation, external fixation, open reduction and internal fixation with a dorsal or volar plating system or a combination of small plate systems[1]. In particular, many clinical reports have demonstrated that internal fixation of unstable distal radial fractures with a volar locking plate system provides excellent outcomes[2-6]. These excellent results are associated with the prevention of radial shorting, malunion, and articular incongruity based on the stable fixation of a volar locking plate system. A number of volar plate systems have been designed and biomechanical studies have reported the stability and ultimate strength of the plates in testing to failure under axial compression[7-10]. The Acu-Loc^® ^Targeted Distal Radius system has recently become available as the volar locking plate which can be characterized by 2 or 3 distal locking screws that target the radial styloid to provide fixation of radial styloid fragments[11]. However, it is unknown whether the radial styloid screws increased the stability of the volar plating system fixation along the entire distal radius. We hypothesized that a significant difference in the biochemical stability of unstable distal radial fractures exists between the volar locking plate fixation with and without the radial styloid screws. The purpose of this study was to evaluate whether the distinctive screws targeting the radial styloid were effective in the stable fixation of distal radial fractures using a cadaver unstable intra-articular fracture model.

## Methods

### Specimen and Preparation

Six matched pairs of fresh-frozen human cadaver wrists, complete from the proximal forearms to the metacarpal bones were procured for this study. The average age at the time of death for the cadavers was 76.8 years (range, 59 - 83). One radius from each matched pair was randomly assigned to each of the 2 volar plate fixation groups.

**Figure 1:**
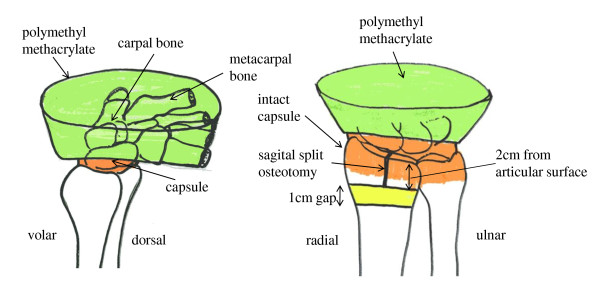
**Wrist full extension and intra-articular unstable fracture model**. Polymethyl methacrylate was mounted on the metacarpal bones of each specimen to simulate axial loading of the distal radius across the intact wrist joint at full wrist extension (A). A standardized 3-part intra-articular and severe comminuted fracture was simulated by making a 1-cm transverse gap at a position 2-cm proximal to the articular surface of the lunate fossa, and a second sagital split osteotomy was performed between the scaphoid and lunate fossa (B).

**Figure 2:**
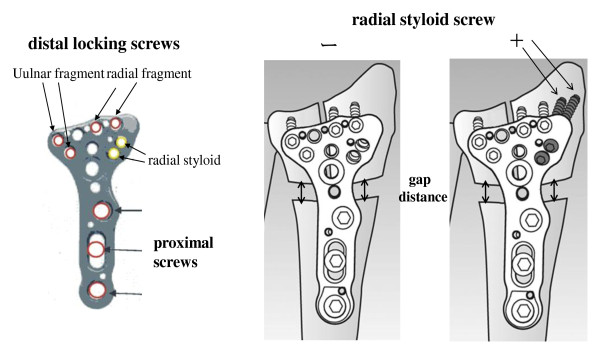
**Acu-Loc^® ^volar plate system**. Two locking screws fixed the ulnar side fracture fragment and 2 locking screws fixed a radial fragment using a Acu-Loc^® ^volar plate system (Acumed, Hillsboro, OR) with (+) or without (-) 2 locking screws targeting the radial styloid, and 3 cortical screws in the proximal fragment. gap distance, gap between the radial or ulnar fragment and the proximal fragment

Specimens were thawed at room temperature on the day of testing. Skin and soft tissues were removed, and the wrist capsule and interosseous membrane, triangular disc, and the capsule of the distal radioulnar joint were left intact. A standardized 3-part intra-articular and severe comminuted fracture was simulated as reported previously with some modification [7,9]. Briefly, a 1-cm transverse gap was made at a point 2-cm proximal to the articular surface of the lunate fossa. A second sagital split osteotomy was performed between the scaphoid and lunate fossa under protection of the wrist and distal radioulnar joints, creating an unstable intra-articular fracture with both radial- and unlar-side fracture fragments. In addition, polymethyl methacrylate was mounted on the metacarpal bones of each specimen to simulate axial loading of the distal radius across the intact wrist at full extension (Figure 1). Specimens were then fixed with the Acu-Loc^® ^volar plate system (Acumed, Hillsboro, OR). Two locking screws were used to fix the ulnar fragment and 2 more to fix the radial fragment, while 2 locking screws and one cortical screw were used to fix the proximal fragment. In addition, the radial fragment was fixed with (+) or without (-) 2 locking screws targeting the radial styloid (Figure 2).

### Biomechanical Testing

**Figure 3:**
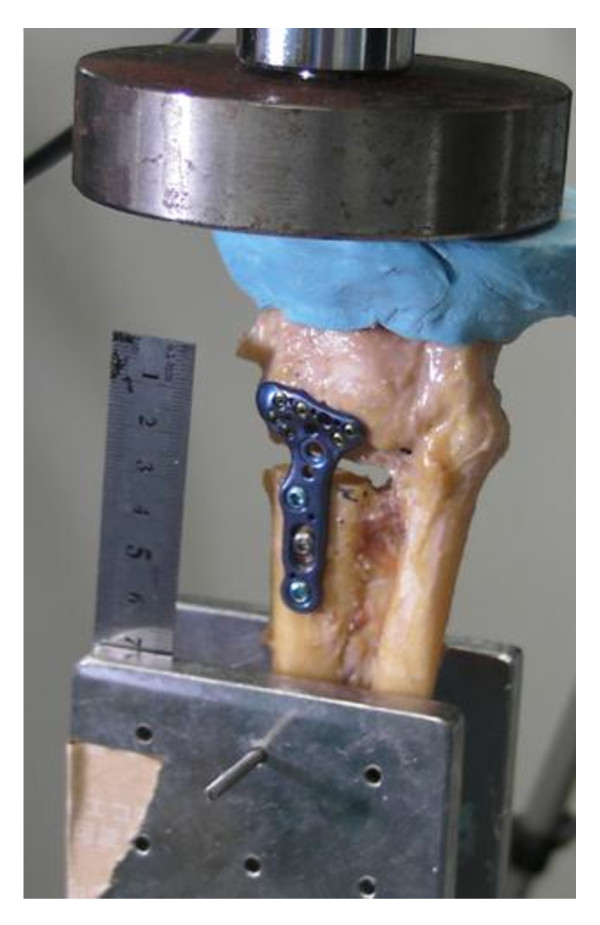
**A Material Testing Machine**. A Material Testing Machine (Autograph, Shimadzu, Kyoto, Japan) load frame was mounted on the flat of polymethyl methacrylate surface on the metacarpal bones of each specimen with the wrist at full extension. Each specimen was loaded at a constant rate of 20 mm/min to failure under compression.

The proximal radius was placed in a Materials Testing Machine (Autograph, Shimadzu, Kyoto, Japan), and a load frame was mounted to the flat surface of the polymethyl methacrylate on the metacarpal bones of specimen at full extension of the wrist (Figure 3). Each specimen was loaded at a constant rate of 20 mm/min to failure. Load data was recorded by a computer and plotted graphically. Ultimate strength was defined as the peak load followed by a sharp decrease in the load-time curve[7]. Gap closing data was recorded using a digital video camera (Digital Movie Camera DMX-HD, Sanyo Ltd, Osaka, Japan). After testing, distal radial bones, fixation plates and screws were examined for signs of failure.

### Statistical Analysis

Data from the 2 groups, fixation with (+) or without (-) the locking screws targeting the radial styloid, were compared. Student's *t *test and Mann-Whitney U test were used to determine the significance of observed differences. A *p *value of less than .05 was considered statistically significant.

## Results

**Figure 4:**
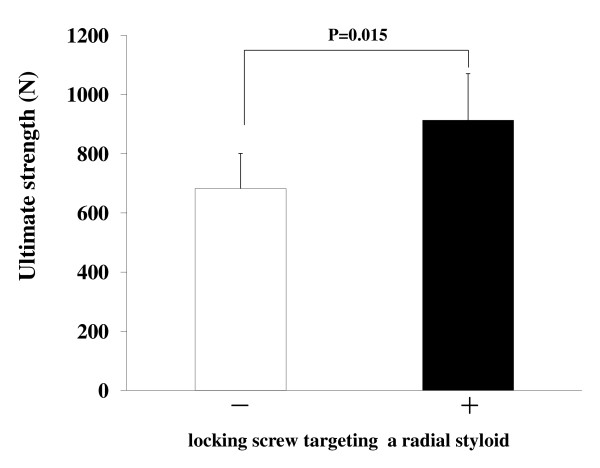
**Comparison of average ultimate strength of volar plate fixation with or without radial styloid screws**. The average ultimate strength of volar plate fixation with radial styloid screws (+; 913.5 ± 157.1 N) (black bar) was significantly higher than that without radial styloid screws (-; 682.2 ± 118.6 N) (white bar).

The average ultimate strength at failure of the volar plate fixation with radial styloid screws (913.5 ± 157.1 N) was significantly higher than that without radial syloid screws (682.2 ± 118.6 N) (Figure 4).

**Figure 5:**
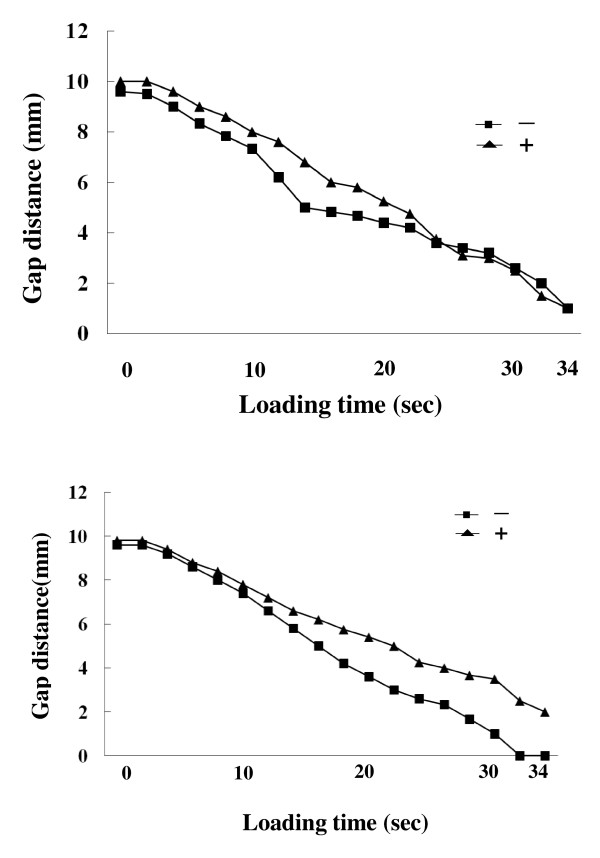
**The average change in gap between the radial and proximal fragment**. **(A)** The average gap in the case of fixation without radial styloid screws (-) tended to decrease at about 10 sec after start of loading compared to that in cases with the screws (+), although the differences were not statistically significant and there was no final difference between the 2 groups. **(B)** The average change in gap between the ulnar and proximal fragment. The average gap in the cases of fixation without radial styloid screws (-) decreased after 10 sec under the loading, compared to that in cases of fixation with the screws (+), though the differences were not significant.

The average change in gap between the radial or ulnar fragment and the proximal fragment (Figure 2) decreased with loading time. The gap distance in cases of fixation without radial styloid screws (-) tended to be lower at about 10 sec after the start of loading compared to that in cases with the screws (+), though there was no final difference between the 2 groups (Figure 5A). On the other hands, the average distance in gap between the ulnar fragment and the proximal fragment in cases of fixation without radial styloid screws (-) was lower after 10 sec under loading, compared to that with screws (+), although the differences were not statistically significant (Figure 5B).

**Figure 6:**
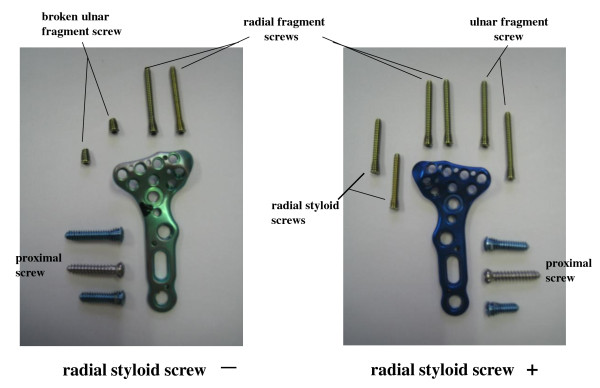
**Example cases of plate and screws after failure of fixation**. When volar plate fixation was performed without radial styloid screws, both ulnar fragment screws were broken while radial fragment screws remained intact (radial styloid screw -). In contrast, both ulnar screws remained intact when volar plate fixation was performed using 2 radial styloid screws (radial styloid screw +).

Figure 6 shows example of plates and screws after the experiments. In cases of volar plate fixation without radial styloid screws, both 2 ulnar fragment screws were broken while the radial fragment screws remained no broken. In contrast, both ulnar screws remained intact in cases of volar plate fixation with 2 radial styloid screws.

**Figure 7:**
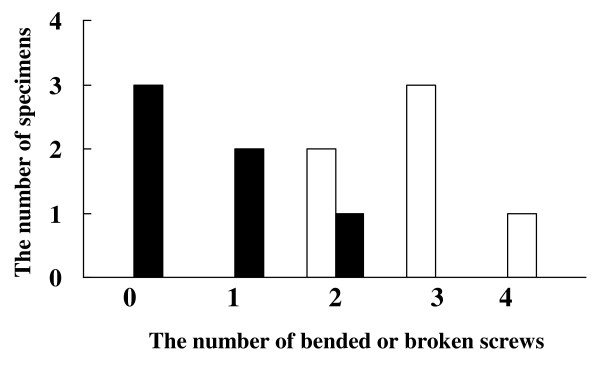
**The number of specimens with bended or broken distal locking screws at the failure of fixation**. The number of specimens with bended or broken screws in the cases of fixation using radial styloid screws (black bar) were significantly fewer than those in the cases not using radial styloid screws (white bar) (Mann-Whitney's U test, p = 0.0065).

**Figure 8:**
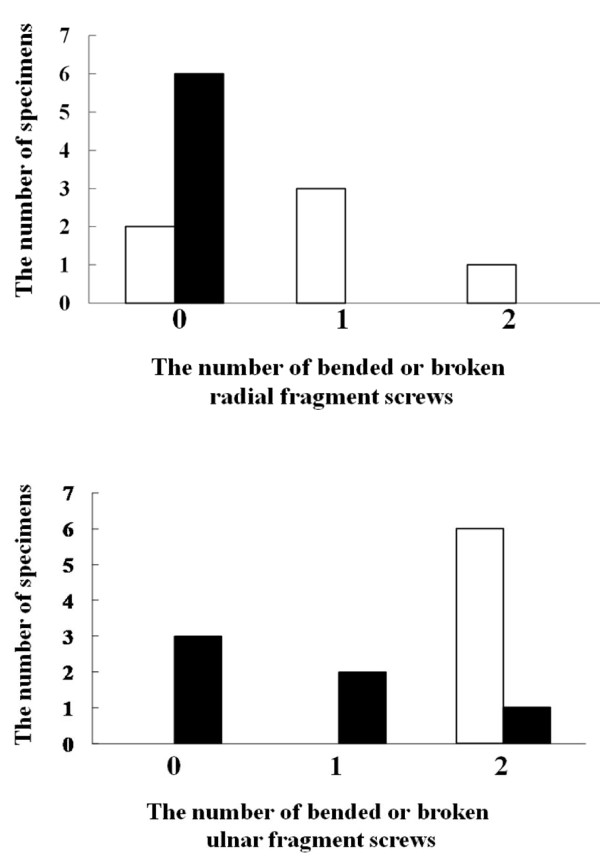
**The number of specimens with bended or broken radial (A) or ulnar (B) fragment screws at the failure of fixation**. There were no bended or broken screws in the cases of fixation with radial styloid screws (black bar) whereas in four of six (66.7%) specimens without radial styloid screw fixation (white bar), 1 or 2 screws were bended or broken (A). Three of six specimens (50%) in the case of fixation with radial styloid screws (black bar) revealed that 2 of 2 screws were intact, and number of specimens with bended or broken screws was significantly fewer in the case of fixation using radial styloid screws (black bar) compared to those not using radial styloid screws (white bar) (B).

After loading to failure, the number of bent or broken screws among the 4 distal screws inserted into the radial and ulnar fragments was examined. In volar plate fixation without radial styloid screws (-), 2 of 4 screws were bent or broken in two specimens, 3 of 4 screws in three specimens, and all 4 screws in one specimen. In volar plate fixation with radial styloid screws, no screws were bent or broken in three four specimens, 1 of 4 screws in two specimens, and 2 of 4 screws in one specimen. Failure loading did not result in any bent or broken volar plates or proximal screws. The number of specimens with bent or broken screws in the fixation using radial styloid screws group was significantly fewer than that in the fixation without radial styloid screws group (Figure 7; Mann-Whitney's U test, p = 0.0065). With regard to the 2 radial fragment screws, there were no bent or broken screws in the fixation with radial styloid screws (+) group, but 1 or 2 screws were bent or broken in four of six (66.7%) specimens without radial styloid screw fixation (-) (Figure 8A). For the 2 ulnar fragment screws, all specimens (100%) revealed both screws to be bent or broken in the fixation without radial styloid screws (-) group, whereas three of six specimens (50%) in the fixation with radial styloid screws (+) group revealed that both screws to be intact, and the number of specimens with bent or broken screws tended to be fewer (Figure 8B).

## Discussion

New developments in vloar plate and locking screw design have improved results of surgical treatment of distal radial fractures,[2-6] and several biomechanical studies have shown that a volar plate and locking screw system is efficient in the stabilizing of fractures against axial force[7-10]. Recently, the Acu-Loc^® ^Targeted Distal Radius system was designed as a best fit at the watershed line with 2 rows of distal locking screws and 1 or 2 screws targeting the radial styloid which theoretically provides greater stability against radial styloid fragments[11]. We undertook biomechanical testing to determine the efficacy of the distinctive screws targeting radial styloid in the stable fixation of entire distal radial fractures using a fresh-frozen human cadaver fracture model.

In the present study, we showed that the radial styloid screws were effective in increasing the ultimate strength at failure of the volar plate fixation (Figure 4) and that their use led to decrease in the number of bent or broken screws after failure loading (Figures 7 and 8). In cases of fixation without radial styloid screws (-), the ulnar fragment was prone to greater displacement than was the fragment with radial styloid screws (+) under axial loading. Interestingly, no difference in the gap amount at the radial fragments was found between fixation with (+) and without (-) the radial styloid screws. Furthermore, in all six specimens without radial styloid screws, both the ulnar fragment screws were broken. On the other hand, only one of six specimens with radial styloid screws revealed both ulnar fragment screws to be broken (Figure 8B). Based on these results, the ulnar fragment appeared to be more intensively stressed than the radial fragment under axial loading of the distal radius at full wrist extension. Previous study showed that force transmission patterns with the wrist in a neutral position consisted of 50% across the scaphoid fossa, 35% across the lunate fossa, and the remaining 15% across the triangular fibrocartilagenous complex [12]. We speculated that there was different pressure distribution under axial loading with the wrist in full extension, although we did not measure pressure in the wrist. Furthermore, we demonstrated that radial styloid screws could significantly increase ulnar fragment stability in cases of volar plate fixation for intra-articular distal radial fracture. Thus, additional fixation using the radial styloid screws was effective in preserving the stability of unstable and intra-articular distal radial fractures. We recommended that the radial styloid screws would be used in volar plate fixation for distal radial fracture regardless of the presence or absence of radial styloid fracture while the additional styloid screw fixation was not critical.

Recent trends in distal radial fracture fixation have emphasized anatomic reduction and rigid fixation allowing early mobilization and return to functional activities. Most previously reported studies directly loaded the isolated radius using a cadaver fracture model;[7,8,10] however, a more clinically relevant loading pattern was that used by Taylor et al[9] in which loading was directed across the wrist joint. In this study, we modified their fracture model so that the positioning of wrist was at full extension, and axial compression was loaded through a flat palmar surface comprised of polymethyl methacrylate on the metacarpal bones. This model better simulated the clinical conditions, such as a fall on an outstretched hand or push-up after internal fixation for intra-articular unstable distal radial fracture.

There are several limitations of this study. First, it seemed to be difficult to decide the failure mode for distal radial fracture based on the small sample size of this study, although the data showed the tendency of failure pattern and would be valuable information to make a plan in fixation of unstable intra-articular fracture. Second, we could not examine the rigidity of the plate system because specimens included several joint spaces and soft tissue connections between joint spaces. Third, the distal radius was loaded across the wrist in an extended position only, not in a flexed nor neutral position. Fourth, we did not examine a cyclic loading model using a physiological load.

## Conclusion

We showed that the distinctive screws targeting the radial styloid were effective in the stable fixation of distal radial fractures in the volar plate and locking screw system (Acu-Loc^® ^volar plate system) using a cadaver unstable intra-articular fracture model.

Acu-Loc^® ^volar plate systems were provided by Acumed, Hillsboro, OR.

## Competing interests

The authors declare that they have no competing interests.

No benefits in any form have been received or will be received from a commercial party related directly or indirectly to the subject of this article. All the authors have no conflicts of interest.

## Authors' contributions

KI, YO and TW carried out the preparation of specimens, establishment of the fracture model and data analysis. The biomechanical experiment and data analysis were carried out by TK and MA. TY and MA participated in the study design and coordination, and helped to draft the manuscript. All authors read and approved the final manuscript.
